# Supplementary material for: An oscillatory mechanism for multi-level storage in short-term memory
Source: Commun Biol. 2023 Aug 10;6:829. doi: 10.1038/s42003-023-05200-7 (PMC10415352; doi:10.1038/s42003-023-05200-7)
Supplement: Supplementary file 2 — Supplementary Information [file 42003_2023_5200_MOESM2_ESM.pdf]

Supplementary Information

Contents

Supplementary Note 1: Model neurons and networks **2**
Description of the Wang-Buzsaki neuron model 2
Networks of Wang-Buzsaki model neurons 3
Simplest network example: the autapse 3
Multi-neuron networks 3
Modified Seung autapse model 4
Phase-locking analysis of the integrate-and-fire neuron model 5

Supplementary Note 2: Stochastic simulations **6**
Networks with time-varying noise in their inputs 6
Networks with (frozen) noise in their synaptic weights 7
Networks with randomized phases of oscillatory input 7

Supplementary Note 3: Parameters specific to simulations **7**

Supplementary References **9**

Supplementary Figures **9**

Supplementary Note 1: Model neurons and networks

In this supplement, we provide a detailed description of the neuronal and network models used in this work. The multistable networks with oscillatory input make use of a conductance-based, single-compartment model neuron described in [3], which we refer to here as the Wang-Buzsaki (W-B) neuron. The equations and parameter values governing the intrinsic response properties of this model, in which spiking is generated through Hodgkin-Huxley-type Na^+ and K^+ voltage-dependent ion currents, are described in the section **Description of the Wang-Buzsaki neuron model** below. In the section **Networks of Wang-Buzsaki model neurons**, we describe the network models based on oscillatory input. In the section **Modified Seung autapse model**, we describe the approximately linear autapse model, adapted from [1], whose robustness to detuning of the autapse strength was compared to that of the W-B autapse model. In the section **Phase-locking analysis of the integrate-and-fire neuron model**, we describe the conditions under which an integrate-and-fire model neuron may be expected to produce the phase-locking (and associated staircase) phenomenon that we have described in the main text.

Description of the Wang-Buzsaki neuron model

The membrane potential of the W-B neuron obeys the current balance equation

$$C_m \frac{\mathrm{d}V}{\mathrm{d}t} = -I_{Na}(V,h) - I_K(V,n) - I_L(V) + I_{syn} + I_{app} \tag{S.1}$$

with kinetics described in [3]:

$$I_{Na}(V,h) = g_{Na} m_\infty^3(V) h (V - E_{Na}) \tag{S.2}$$

$$I_K(V,n) = g_K n^4 (V - E_K) \tag{S.3}$$

$$I_L(V) = g_L (V - E_L) \tag{S.4}$$

$$\tau_h(V)\frac{\mathrm{d}h}{\mathrm{d}t} = \phi\left(h_\infty(V) - h\right), \quad \tau_n(V)\frac{\mathrm{d}n}{\mathrm{d}t} = \phi(n_\infty(V) - n) \tag{S.5}$$

$$m_\infty(V) = \frac{\alpha_m(V)}{\alpha_m(V)+\beta_m(V)}, \quad h_\infty(V) = \frac{\alpha_h(V)}{\alpha_h(V)+\beta_h(V)}, \quad n_\infty(V) = \frac{\alpha_n(V)}{\alpha_n(V)+\beta_n(V)} \tag{S.6}$$

$$\tau_h(V) = \frac{1}{\alpha_h(V)+\beta_h(V)}, \quad \tau_n(V) = \frac{1}{\alpha_n(V)+\beta_n(V)} \tag{S.7}$$

$$\alpha_m(V) = 0.1\frac{V+35}{1-\exp(-0.1(V+35))}, \quad \beta_m(V) = 4\exp\left(-\frac{V+60}{18}\right) \tag{S.8}$$

$$\alpha_h(V) = 0.07\exp\left(-\frac{V+58}{20}\right), \quad \beta_h(V) = \frac{1}{\exp(-0.1(V+28))+1} \tag{S.9}$$

$$\alpha_n(V) = 0.01\frac{V+34}{1-\exp(-0.1(V+34))}, \quad \beta_n(V) = 0.125\exp\left(-\frac{V+44}{80}\right) \tag{S.10}$$

The synaptic and applied currents, I_{syn} and I_{app}, respectively, are specified for different model architectures in the following sections. The common parameters used for all simulations involving W-B model neurons are specified in Supplementary Table 1. We defined a spike to have occurred when the voltage crossed a voltage threshold of 0 mV in the upward direction and defined the spike times as occurring at the time of the peak of the membrane potential V subsequent to theshold crossing. Integration was performed numerically using the fourth-order Runge-Kutta method with time step $\Delta t = 10^{-2}$ ms.

$g_{Na} = 35$ mS/cm^2	$E_{Na} = 55$ mV	$\phi = 15$
$g_K = 9$ mS/cm^2	$E_K =$ -90 mV	$C_m = 0.333$ μF/cm^2
$g_L = 0.5$ mS/cm^2	$E_L =$ -65 mV	

Supplementary Table 1: Parameters common to all W-B neuron simulations

Non-oscillatory input	Oscillatory input
$w_{syn} = 5.5\ \mu\text{A/cm}^2$<br>$I_0 = 3.515\ \mu\text{A/cm}^2$<br>$\psi = 0\ \mu\text{A/cm}^2$<br>$\tau_{syn} = 150$ ms	$w_{syn} = 5.5\ \mu\text{A/cm}^2$<br>$I_0 = 3.515\ \mu\text{A/cm}^2$<br>$\psi$ = -0.5 $\mu\text{A/cm}^2$<br>$\tau_{syn} = 150$ ms<br>$\omega = 0.05\ \text{ms}^{-1}$ (unless specified otherwise)

Supplementary Table 2: Parameters specific to the single-neuron (autapse) networks

Networks of Wang-Buzsaki model neurons

The different network architectures used in this work are described in the following subsections. The simplest of these, a single model neuron recurrently connected to itself (the autapse model), is described in the subsection **Simplest network example: the autapse**. In the subsection **Multi-neuron networks**, we describe the multi-neuron cases of all-to-all connected networks, a ring network, and a network that exhibits sequential drift of a bump of activity. The parameters specific to the autapse network simulations are listed in Supplementary Table 2 and those specific to multi-neuron networks are listed in Supplementary Table 3. Equations and parameter values for the stochastic simulations of multi-neuron networks are provided in **Supplementary Note 2: Stochastic simulations**.

Simplest network example: the autapse

The autapse model consists of a W-B model neuron recurrently connected to itself and is described as follows:

$$C_m \frac{\mathrm{d}V}{\mathrm{d}t} = -I_{Na}(V,h) - I_K(V,n) - I_L(V) + I_{syn}(s) + I_0 + I_{osc}(t) + I_{ext}(t) \tag{S.11}$$

$$I_{syn}(s) = w_{syn} s \tag{S.12}$$

$$\tau_{syn} \frac{\mathrm{d}s}{\mathrm{d}t} = -s + \alpha_{syn} \sum_{t^{spike}} \delta(t - t^{spike}) \tag{S.13}$$

$$I_{osc}(t) = \psi \cos(\omega t) \tag{S.14}$$

The neuron receives several sources of inputs: a recurrent synaptic feedback current $I_{syn}(s)$, the strength of which is determined by w_{syn}; a DC current offset I_0; an oscillatory input current $I_{osc}(t)$ (set to zero for simulations with no oscillatory input); and a current corresponding to the external stimulus $I_{ext}(t)$. The parameters specific to oscillatory and non-oscillatory simulations are listed in Supplementary Table 2. $\alpha_{syn} = 1.0$ ms for all simulations.

Multi-neuron networks

For the networks composed of multiple W-B neurons, each individual neuron i is governed by the same equations as for the single neuron case, except that the synaptic current input $I_{syn,i}(s_1, ..., s_N)$ depends on the activity of all other neurons j of the network through the synaptic weights w_{ij}:

$$C_m \frac{\mathrm{d}V_i}{\mathrm{d}t} = -I_{Na}(V_i,h_i) - I_K(V_i,n_i) - I_L(V_i) + I_{syn,i}(s_1, ..., s_N) + I_0 + I_{osc,i}(t) + I_{ext,i}(t) \tag{S.15}$$

$$I_{syn,i}(s_1, ..., s_N) = \sum_{j=1}^{N} w_{ij} s_j \tag{S.16}$$

$$\tau_{syn} \frac{\mathrm{d}s_i}{\mathrm{d}t} = -s_i + \alpha_{syn} \sum_{t_i^{spike}} \delta(t - t_i^{spike}) \tag{S.17}$$

For all networks presented in this paper, we used a value of $\alpha_{syn} = 1.0$ ms.

Non-oscillatory input	Oscillatory input
τ_{syn} = 150 ms<br>I_0 = 3.515 μA/cm^2 (fully connected network)<br>I_0 = 4.01 μA/cm^2 (ring, sequential activity networks)<br>ψ = 0 μA/cm^2	τ_{syn} = 150 ms, 80 ms (Supp Fig 1c), 30 ms (Supp Fig 1a,b)<br>I_0 = 3.515 μA/cm^2<br><br>ψ = -0.5 μA/cm^2<br>ω = 0.05 ms^{-1} (unless specified otherwise)

Supplementary Table 3: Parameters specific to the multi-neuron networks of W-B neurons

Fully connected network. In Figure 6 and Supplementary Figures 1 and 3, we implemented fully connected (all-to-all) networks of $N = 1000$ W-B neurons. Except for the stochastic simulations described in **Supplementary Note 2: Stochastic simulations**, the connection strength from neuron j to neuron i was given by:

$$w_{ij} = \frac{5.5}{N}\ [\mu\text{A}/cm^2] \tag{S.18}$$

Ring network. For the ring network illustrated in Figure 7 ($N = 100$ neurons), the connection strength from neuron j to neuron i was described by a symmetric, rotationally invariant connection matrix:

$$w_{ij} = A + B\cos\left(\frac{2\pi(i-j)}{N}\right)\ [\mu\text{A}/cm^2]. \tag{S.19}$$

$A = -0.024$ [μA/cm^2] and $B = 0.059$ [μA/cm^2] for the non-oscillatory case, and $A = -0.54$ [μA/cm^2] and $B =$ 0.909 [μA/cm^2], with $\psi = 0.5$ μA/cm^2 for the oscillatory case.

Sequential activity network. In Figure 8, we illustrate an asymmetrically connected network ($N = 100$ neurons), which resembles the ring network, but whose asymmetry enables a well-controlled drift of the bump attractor in one direction. The connection strength from neuron j to neuron i was defined by:

$$w_{ij} = A + B\cos\left(\frac{4\pi(i-j)}{N} + 0.1\right)\mathcal{H}\left(C - |i-j|\right)\ [\mu\text{A}/cm^2], \tag{S.20}$$

where $\mathcal{H}$ denotes the Heaviside (step) function. $C = 38$ for all simulations. $A = -0.2$ [μA/cm^2] and $B = 0.303$ [μA/cm^2] for the simulations with no oscillatory input, and $A = -1.08$ [μA/cm^2] and $B = 1.818$ [μA/cm^2] for the simulations with oscillatory input.

Modified Seung autapse model

In Figure 5c,d, we simulated a modified version of the model described in [1] (referred to here as the Seung autapse model) in order to compare its robustness to that of the W-B autapse model with oscillatory input. The intrinsic neuron parameters are based on the model introduced by Shriki and colleagues [2], which exhibits an approximately linear f-I curve due to the inclusion of an A-type potassium current ($I_A(V_k, b_k)$ in the equations below). The model receives feedback current through a synapse onto itself (autapse). The model equations are:

$$C_m\frac{\mathrm{d}V}{\mathrm{d}t} = -I_{Na}(V,h) - I_K(V,n) - I_L(V) - I_A(V,b) + I_E + I_{ext} \tag{S.21}$$

$$I_E(V) = (w_S S + g_O)(V - E_E) \tag{S.22}$$

g_{Na} = 100 mS/cm^2	E_{Na} = 55 mV	$\alpha_s = 1$	C_m = 1 μF/cm^2
g_K = 40 mS/cm^2	E_K = -80 mV	θ_s = -20 mV	$\phi = 10$
g_L = 0.2 mS/cm^2	E_L = -65 mV	σ_s = 2 mV	τ_A = 20 ms
g_A = 20 mS/cm^2	E_E = 0 mV	w_S = 1.882 mS/cm^2	τ_S = 100 ms
g_O = 0.0353 mS/cm^2			

Supplementary Table 4: Parameters of the modified Seung autapse model

where the kinetics are as described in [1]:

$$I_{Na}(V,h) = g_{Na} m_\infty^3(V) h (V - E_{Na}) \tag{S.23}$$

$$I_K(V,n) = g_K n^4 (V - E_K) \tag{S.24}$$

$$I_L(V) = g_L(V - E_L) \tag{S.25}$$

$$I_A(V,b) = g_A a_\infty^3(V) b (V - E_K) \tag{S.26}$$

$$\tau_A \frac{\mathrm{d}b}{\mathrm{d}t} = b_\infty(V) - b \tag{S.27}$$

$$\tau_S \frac{\mathrm{d}S}{\mathrm{d}t} = -S + \alpha_S(1-S)\sigma_k(V) \tag{S.28}$$

$$\tau_h(V)\frac{\mathrm{d}h}{\mathrm{d}t} = \phi(h_\infty(V) - h), \quad \tau_n(V)\frac{\mathrm{d}n}{\mathrm{d}t} = \phi(n_\infty(V) - n) \tag{S.29}$$

$$m_\infty(V) = \frac{\alpha_m(V)}{\alpha_m(V)+\beta_m(V)}, \quad h_\infty(V) = \frac{\alpha_h(V)}{\alpha_h(V)+\beta_h(V)}, \quad n_\infty(V) = \frac{\alpha_n(V)}{\alpha_n(V)+\beta_n(V)} \tag{S.30}$$

$$\tau_h(V) = \frac{1}{\alpha_h(V)+\beta_h(V)}, \quad \tau_n(V) = \frac{1}{\alpha_n(V)+\beta_n(V)} \tag{S.31}$$

$$\alpha_m(V) = 0.1\frac{V+30}{1-\exp(-0.1(V+30))}, \quad \beta_m(V) = 4\exp\left(-\frac{V+55}{18}\right) \tag{S.32}$$

$$\alpha_h(V) = 0.07\exp\left(-\frac{V+44}{20}\right), \quad \beta_h(V) = \frac{1}{\exp(-0.1(V+14))+1} \tag{S.33}$$

$$\alpha_n(V) = 0.01\frac{V+34}{1-\exp(-0.1(V+34))}, \quad \beta_n(V) = 0.125\exp\left(-\frac{V+44}{80}\right) \tag{S.34}$$

$$a_\infty(V) = \frac{1}{\exp\left(-\frac{V+50}{20}\right)+1}, \quad b_\infty(V) = \frac{1}{\exp\left(\frac{V+80}{6}\right)+1} \tag{S.35}$$

$$\sigma(V) = \frac{1}{1+\exp\left(-\frac{V-\theta_s}{\sigma_s}\right)} \tag{S.36}$$

Individual parameters are listed in Supplementary Table 4.

We defined a spike to have occurred when the membrane potential crossed a threshold of 20 mV in the upward direction and defined the spike times as occurring at the time of the membrane potential crossing 0 mV subsequent to theshold crossing. Integration was performed numerically using the fourth order Runge-Kutta method with time step $\Delta t = 2 \cdot 10^{-3}$ ms.

Phase-locking analysis of the integrate-and-fire neuron model

In Figure 4 of the main text, we show that the phase-locking and associated staircase of firing rate versus synaptic input occur in a leaky, but not a non-leaky integrate-and-fire neuron receiving oscillatory input. The key results were that: (1) phase differences caused by a transient pulse of input decay away exponentially during the subthreshold period in the leaky, but not the non-leaky, integrate-and-fire neuron, and (2) the leaky, but not the non-leaky, integrate-and-fire neuron displays the staircase of firing rate versus synaptic input wherein small differences in synaptic input on a given step of the staircase do not cause corresponding changes in firing rate (i.e., # of spikes per cycle). These properties can be seen analytically by examining the differences ΔV between the voltage responses of two integrate-and-fire neuron simulations

defined by the following equations:

$$\frac{dV_1}{dt} = -\frac{1}{\tau}(V_1 - V_L) + I(t) + I_0 + I_{syn_1} + I_{pulse}(t) \quad \text{(S.37)}$$

$$\frac{dV_2}{dt} = -\frac{1}{\tau}(V_2 - V_L) + I(t) + I_0 + I_{syn_2} \quad \text{(S.38)}$$

$$\frac{d\Delta V}{dt} = -\frac{1}{\tau}(\Delta V) + \Delta I_{syn} + I_{pulse}(t) \quad \text{(S.39)}$$

where $\Delta V = V_1 - V_2$ and $\Delta I_{syn} = I_{syn_1} - I_{syn_2}$. Here, as in Eq. 10 of the main text, I(t) represents the oscillatory input, I_0 represents constant background input, and I_{syn} represents the synaptic input. In the simulations of the response to a pulse of input (Figs. 4b,e), $I_{pulse}(t)$ represents the brief pulse of positive (red trace) or negative (orange trace) input and the synaptic input is identical for each simulation ($I_{syn_1} = I_{syn_2}$)

For the non-leaky integrate-and-fire neuron, $\tau \to \infty$ so the first, exponential decay governing term in the voltage difference equation (Eq. S.39) is zero. As a result, for the simulations of Fig. 4b with a brief pulse of input, the voltage difference and corresponding phase shift caused by the pulse of input persists indefinitely. For the simulations of Fig. 4c, in which the two simulations have different synaptic inputs ($\Delta I_{syn} \neq 0$), this leads to a steady accumulation (i.e., temporal integration) of voltage difference between the two (magenta and cyan) traces in the subthreshold dynamics, and a corresponding difference in firing rates.

For the leaky integrate-and-fire neuron, voltage differences in the subthreshold dynamics decay away with time constant τ. Thus, for the simulation of Fig. 4e with a brief pulse of input, the pulse causes only a transient change in firing that decays away exponentially during the subthreshold portion of the oscillation. As long as the exponential decay time constant is much less than the subthreshold period between spiking events, the voltage difference will decay away nearly completely (Fig. 4e). We note that, during the spiking that occurs near the peak of the external oscillation, the voltage differences also exhibit exponential decay between spikes, but the voltage reset caused by the spike then separates the voltage traces from each other and switches which neuron's voltage is higher, so that the phase differences do not steadily decay away during this period of rapid spiking. For the simulations with different synaptic inputs ($\Delta I_{syn} \neq 0$), the exponential decay dynamics lead to a steady-state offset in voltage $\Delta V \to \Delta I_{syn}$. This leads to the observed relative phase shift of the pink and cyan traces in Fig. 4f. However, as long as the voltage difference caused by the different synaptic inputs is not too large, it can phase shift the timing of spikes without permitting the addition of an extra spike; as a result, there is a constant 'plateau' in the firing rate for different levels of synaptic input. For larger synaptic input differences, an extra spike will be added, corresponding to the stepping up between plateaus of the firing rate versus synaptic input staircase.

Supplementary Note 2: Stochastic simulations

For the multi-neuron networks, we considered the effects of several forms of stochasticity: simulations with time-varying random noise in the inputs (the section **Networks with time-varying noise in their inputs**), simulations with "frozen" (not time-varying) noise in the synaptic weights of the network (the section **Networks with (frozen) noise in their synaptic weights**), and simulations in which the phases of the oscillatory external input to different neurons were randomized (the section **Networks with randomized phases of oscillatory input**). We describe our methods for each of these cases below.

Networks with time-varying noise in their inputs

In Figure 6c and Supplementary Figure 3b, we simulated fully connected networks of 1000 W-B neurons with two different types of time-varying noise: the addition of exponentially filtered Gaussian noise, $n_i(t)$, to the input (Figure 6c), or the inclusion of multiplicatively filtered noise in the amplitude and frequency parameters ($\psi(t)$ and $\omega(t)$, respectively) of the oscillatory input $I_i(t)$ (Suppementary Figure 3b). In all cases, the noise was independent for each neuron in the network. The resulting models are described by:

$$C_m \frac{dV_i}{dt} = -I_{Na}(V_i, h_i) - I_K(V_i, n_i) - I_L(V_i) + I_{syn,i}(s_1, ..., s_N) + I_0 + I_{osc,i}(t) + I_{ext,i}(t) + n_i(t) \quad \text{(S.40)}$$

$$I_{osc,i}(t) = \psi_i(t)\cos(\omega_i(t)t) \quad \text{(S.41)}$$

where $n_i(t)$, $\psi_i(t)$, and $\omega_i(t)$ vary in time, as described below.

Noisy external input $n_i(t)$

For the simulations illustrated in Figure 6c, exponentially filtered white noise (an Ornstein-Uhlenbeck process) input was implemented for each individual neuron i as:

$$n_i(t) = n_{i,t-\Delta t} - n_{i,t-\Delta t}\frac{\Delta t}{\tau_n} + \sigma_n \eta_{i,t}\sqrt{\frac{\Delta t}{\tau_n}} \tag{S.42}$$

$$\eta_{i,t} \sim \mathcal{N}(0,1) \tag{S.43}$$

with $\sigma_n = 0.05\ \mu\text{A ms}^{0.5}/\text{cm}^2$.

Noisy oscillatory parameters $\psi_i(t)$ and $\omega_i(t)$

For the simulations illustrated in Supplementary Figure 3b, the amplitude (ψ_i) and frequency (ω_i) of the oscillatory input to each neuron evolved over time through multiplicative noise chosen independently for each neuron i of the network:

$$\psi_i(t) = \bar{\psi} n_{i,\psi,t} \tag{S.44}$$

$$\omega_i(t) = \bar{\omega} n_{i,\omega,t} \tag{S.45}$$

$$n_{i,\psi,t} = n_{i,\psi,t-\Delta t} + (1 - n_{i,\psi,t-\Delta t})\frac{\Delta t}{\tau_\psi} + \sigma_\psi \eta_{i,\psi,t}\sqrt{\frac{\Delta t}{\tau_\psi}} \tag{S.46}$$

$$n_{i,\omega,t} = n_{i,\omega,t-\Delta t} + (1 - n_{i,\omega,t-\Delta t})\frac{\Delta t}{\tau_\omega} + \frac{a\sigma_\omega}{t}\eta_{i,\omega,t}\sqrt{\frac{\Delta t}{\tau_\omega}} \tag{S.47}$$

$$\eta_{i,x,t} \sim \mathcal{N}(0,1) \text{ for x=}\psi,\omega \tag{S.48}$$

with parameters $\bar{\psi} = -0.5\ \mu\text{A/cm}^2$, $\sigma_\psi = 0.025$, $\tau_\psi = 500$ ms, $\bar{\omega} = 0.05\ \text{ms}^{-1}$, $a = 1$ ms, $\sigma_\omega = 0.01$, and $\tau_\omega = 500$ ms, with t in ms. Here, the deterministic portion of each noise equation represents an exponential decay towards a value of 1 and, in the final term of the equation for $n_{i,\omega,t}$, the 1/t cancels the factor of t within the cosine of Eq. S.14.

Networks with (frozen) noise in their synaptic weights

In the simulations illustrated in Figure 6d, the synaptic weights of the networks were initialized by independently adding random noise to the uniform synaptic weights. The random values were independently drawn from a normal distribution with $\mu = 0$ [$\mu\text{A/cm}^2$] and standard deviation $\sigma = 0.055$ [$\mu\text{A/cm}^2$]. The value of the standard deviation was chosen to correspond to approximately where the network's ability to maintain multi-level persistent activity became substantially degraded. This can be thought of as an approximate tolerance value of the 1000-neuron network to variation in the synaptic weights.

Networks with randomized phases of oscillatory input

In Supplementary Figures 1 and 3a, we demonstrate how the distribution of relative phases of oscillatory inputs affects the synaptic time constant needed to maintain multi-level persistent activity. The individual phases of the inputs to the networks illustrated in Supplementary Figures 1b and 3a were initialized by drawing randomly and independently from a uniform distribution on $[0, 2\pi)$. In Supplementary Figure 1c, the phases were initialized by drawing randomly and independently from a von Mises distribution with $\mu = 0$ and concentration parameter $k = 1.0$. In all cases, the networks consisted of 1000 homogeneously connected W-B neurons.

Supplementary Note 3: Parameters specific to simulations

Any parameter values used to generate figures included in the text or Supplementary Information that were not stated above or in the figures are listed below. For consistency, stimulus onsets in simulations that used oscillatory inputs always occurred at the same phase of the oscillation. The parameters used for the simulations of Figs. 7, 8, and Supplementary Figure 3 are depicted in those figures.

Figure 1

All external input durations were 50 ms. The amplitudes of the pulses used to generate Figure 1c were [40 (green), 120 (purple), 164 (orange), 172 (red), 190 (blue)] Hz. The times of stimulus onset in Figure 1e were [100, 2000, 4000, 6000, 8000] ms. The amplitudes of the pulses used to generate Figure 1e (orange traces) were [70, 70, 70, -20, -20] Hz. The amplitudes of the pulses used to generate Figure 1e (blue traces) were [50, 50, 50, -40, -40] Hz. The amplitudes of the pulses used to generate Figure 1e (red traces) were [30, 30, 30, -130, -130] Hz.

Figure 2

All external input durations were 100 ms and the time of stimulus onset was t=62.8 ms. The amplitudes of the pulses used to generate Figure 2b were [0.011 (green), 0.015 (purple), 0.0175 (orange), 0.13625 (red), 0.2 (blue)] $\mu A/cm^2$. The amplitudes of the pulses used to generate Figures 2d,e,f were [0.05 (green), 0.2 (purple), 0.3 (orange), 0.45 (red), 0.6 (blue)] $\mu A/cm^2$.

Figure 3

For Figure 3b, the model was brought to a steady firing rate of 2 spikes/cycle (2 spikes per one cycle of the external oscillatory input) using the same procedure as described above for Figure 2. The excitatory and inhibitory perturbation current-injection pulses occurred at a phase of 1.9 rad (with respect to the external oscillatory input) with duration of 10 ms and amplitude of +/- 0.06 $\mu A/cm^2$. For Figure 3c, the synaptic activation was held at a constant value of 0.012 (top) or 0.019 (bottom).

Figure 4

For each simulation shown in Figure 4, the synaptic input $I_{syn} = w_{syn}s$ was held constant, with $w_{syn} = 16.5$ $\mu A/cm^2$. For Figures 4b,e, 10 ms pulses occurring at simulated time $t = 1415$ ms were used for perturbations. For Figure 4b, $s = 0.0321$ and pulses had amplitude of -0.24 and 0.325 $\mu A/cm^2$. For Figure 4e, $s = 0.014$ and pulses had amplitude of -0.325 and 0.325 $\mu A/cm^2$. For Figure 4c, $s = 0.02815$ or 0.02833. For Figure 4f, $s = 0.012$ or 0.02.

Figure 5

All external input durations were 100 ms for Figure 5a and 50 ms for Figure 5c; the times of stimulus onset were [62.8, 1068.1, 2073.5, 3078.8, 4084.1, 5089.4, 6094.7, 7100.0, 7979.6, 8985.0] ms. The amplitudes of the pulses used to generate Figure 5a were [0.2, 0.1125, 0.15, -0.225, 0.225, -0.225, -0.1125, 0.15, -0.15, 0.1125] $\mu A/cm^2$. The amplitudes of the pulses used to generate Figure 5c were [2.0, 0.75, 1.0, -1.5, 1.5, -1.5, -0.75, 1.0, -1.0, 0.75] $\mu A/cm^2$.

Figure 6

All external input durations were 100 ms and the times of stimulus onset were [62.8, 1947.8, 3958.4, 5969.0, 7979.6, 9990.3, 11875.2, 13885.8, 15896.5, 17907.1] ms. The amplitudes of the pulses used to generate Figure 6b were [0.1125, 0.125, 0.1375, 0.135, 0.175, 0.175, 0.175, -0.175, -0.175, -0.175] $\mu A/cm^2$. The amplitudes of the pulses used to generate Figure 6c were [0.1, 0.125, 0.125, 0.1375, 0.175, 0.175, 0.2, -0.175, -0.175, -0.175] $\mu A/cm^2$. The amplitudes of the pulses used to generate Figure 6d were [0.1, 0.125, 0.125, 0.125, 0.125, 0.125, 0.125, -0.175, -0.175, -0.175] $\mu A/cm^2$.

Supplementary Figure 1

All external input durations were 50 ms and the times of stimulus onset were [62.8, 2073.5, 4084.1, 6094.7, 8105.3, 10115.9] ms. The amplitudes of the pulses used to generate Supplementary Figure 1a were [0.05, 0.1, 0.15, 0.2, 0.25, 0.3] $\mu A/cm^2$. The amplitude of all pulses used to generate Supplementary Figure 1b was 0.03125 $\mu A/cm^2$. The amplitude of all pulses used to generate Supplementary Figure 1c was 0.075 $\mu A/cm^2$.

Supplementary Figure 2

All external input durations were 100 ms and the times of stimulus onset were [62.8, 1947.7, 3958.4, 5969.0, 7979.6, 9990.2, 11875.2] ms. The amplitudes of the pulses were [0.1, 0.125, 0.125, 0.1375, -0.125, -0.125, -0.125] $\mu A/cm^2$. The nonzero connection weights had strength 0.0022 $\mu A/cm^2$.

Supplementary References

[1] H. Sebastian Seung, Daniel D. Lee, Ben Y. Reis, and David W. Tank. The autapse: a simple illustration of short-term analog memory storage by tuned synaptic feedback. *Journal of Computational Neuroscience*, 9(2):171–185, 2000.

[2] Oren Shriki, David Hansel, and Haim Sompolinsky. Rate models for conductance-based cortical neuronal networks. *Neural Computation*, 15(8):1809–1841, 2003.

[3] Xiao-Jing Wang and György Buzsáki. Gamma oscillation by synaptic inhibition in a hippocampal interneuronal network model. *Journal of Neuroscience*, 16(20):6402–6413, 1996.

Supplementary Figures

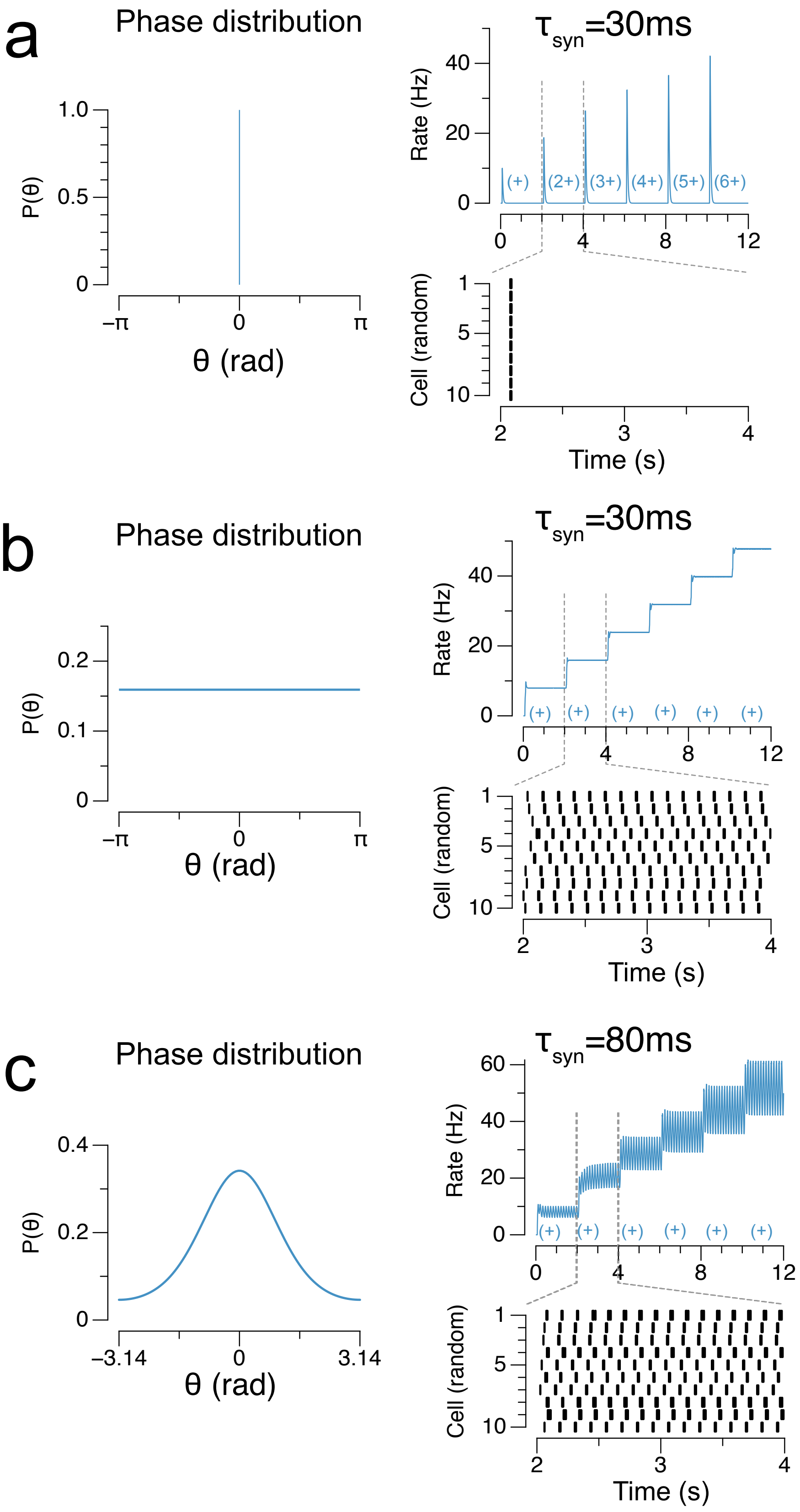

Supplementary Figure 1. Heterogeneity in the phases of oscillations in the network enables discretely graded persistent activity using synaptic time constants that are much shorter than the oscillation cycle. (a) Failure of network with perfectly aligned phases and shorter synaptic time constants to maintain persistent activity. When all oscillatory phases are perfectly aligned (left), the currents provided by the shorter time constant synapses (τ_{syn} = 30 ms) cannot bridge the troughs of the oscillation cycles; as a result, the network fails to maintain persistent firing (right). Times and sizes of external pulses of increasing magnitude are depicted along the x-axis with (n+) denoting a pulse of n times the magnitude of (+). (b) Distributed phases of oscillatory inputs enables the network to store discretely graded activity even with 30 ms synaptic time constants. When the phases of the oscillatory inputs to the network are independently drawn from a uniform distribution (left), the relative spread in timing of spiking responses in the network can bridge the oscillation cycles and enable discretely graded persistent activity. (c) Network response to a distribution of oscillatory inputs intermediate between the phase distributions illustrated in panels a and b. Left, distributions were generated by sampling the phases from a Von Mises distribution (concentration parameter k=1.0). Right, the network can maintain discretely graded persistent activity with synaptic time constants (τ_{syn} = 80 ms shown here) typically used in models of persistent activity.

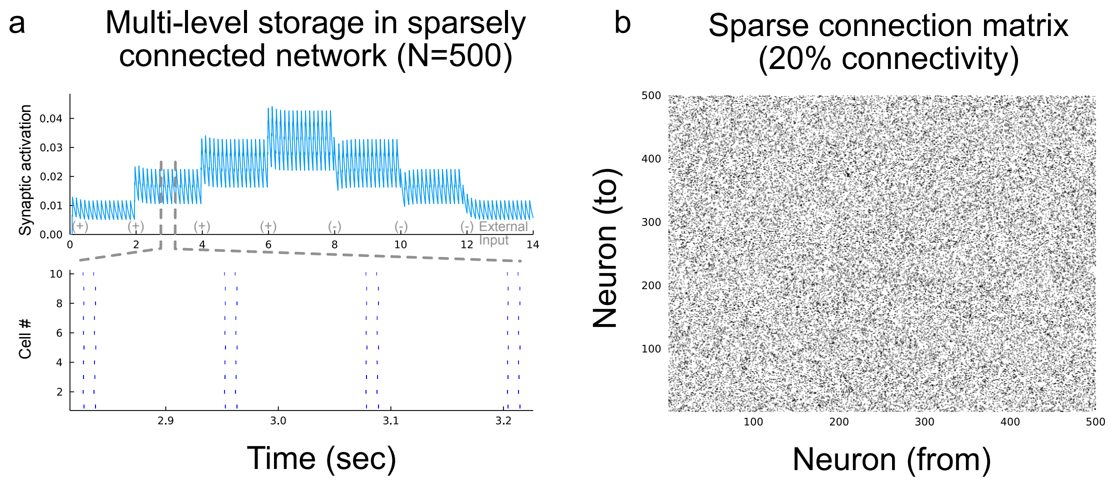

Supplementary Figure 2. Multi-level storage of persistent neural activity in a sparsely connected network (N=500 neurons). (a) Mean firing rate across the population (top) and rasters of individual neuron responses (bottom, axis expanded to show 4 cycles). The input to the network is a sequence of brief (100 ms) external input pulses (approximate times of positive and negative pulses denoted in gray along the x-axis). (b) Plot of network connection weights (black = connected, white = not connected); each neuron was randomly connected to 100 other neurons in the network. The network size of 500 neurons (rather than 1000) was chosen here for easier visualization of the connectivity matrix.

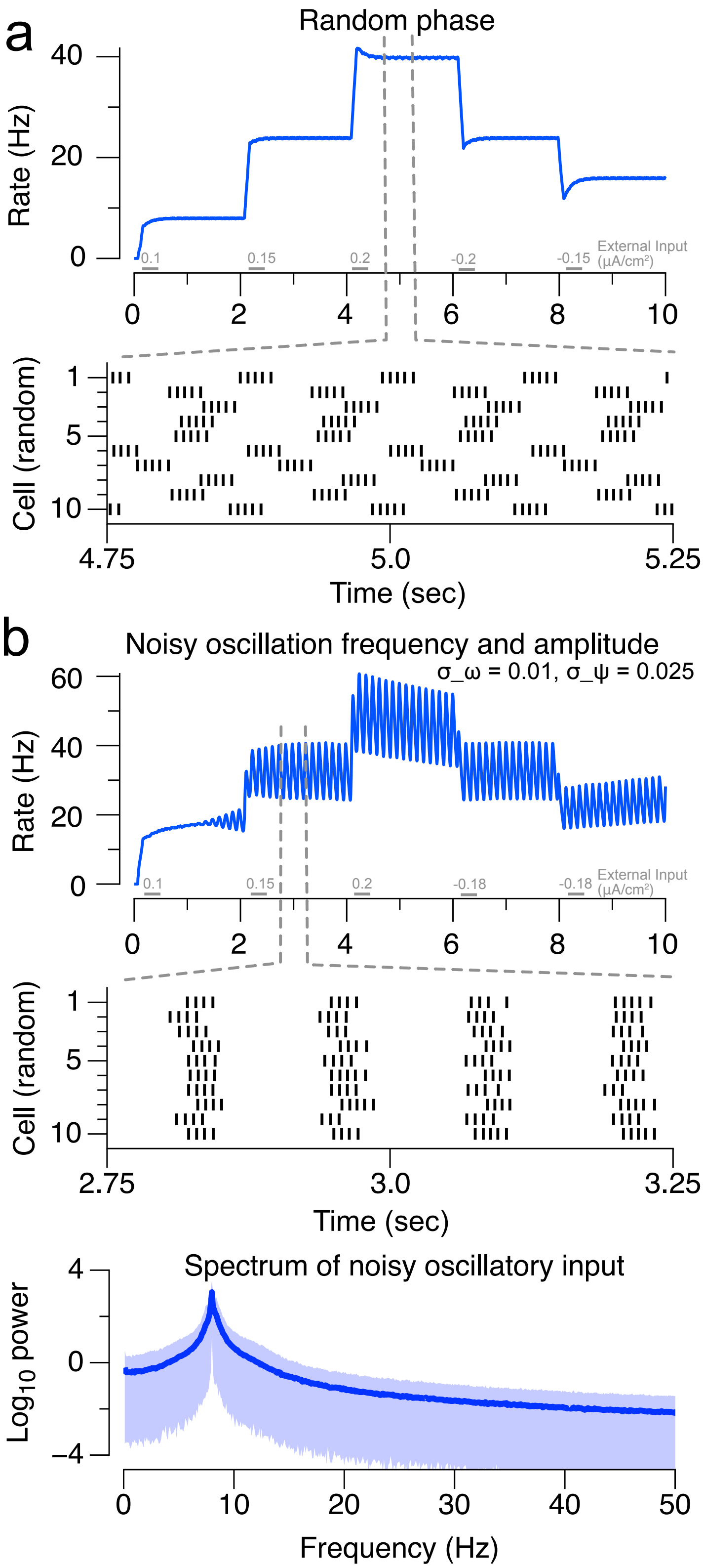

Supplementary Figure 3. Robustness of persistent activity in a fully connected network of 1000 WB neurons receiving noisy oscillatory input or oscillatory input with randomized phase across neurons. (a) Mean firing rate across the population (top) and rasters of individual neuron responses (bottom) for a network in which the phase of oscillatory input is randomly chosen from a uniform distribution for each neuron. Input to the network is a sequence of brief (100 ms) input pulses (gray bars). (b) Mean firing rate across the population (top) and rasters of individual neuron responses (middle) to a sequence of brief input pulses in a network in which the values of the oscillatory parameters ω and ψ vary randomly in time (Supplementary Note 2). Bottom, power spectrum of the oscillatory input. Noise is independent (uncorrelated) across neurons of the network. Shading corresponds to 95% confidence intervals.
